# Pseudoaneurysms: a rare complication of infective endocarditis in an adolescent with bicuspid aortic valve

**DOI:** 10.1093/ehjcr/ytaf270

**Published:** 2025-05-28

**Authors:** Manuela Lopes, Sofia Rito, David Prieto, António Pires

**Affiliations:** Department of Pediatric Cardiology, Referral Center for Congenital Cardiac Defects, Unidade Local de Saúde de Coimbra, 3000-602 Coimbra, Portugal; Department of Pediatric Cardiology, Referral Center for Congenital Cardiac Defects, Unidade Local de Saúde de Coimbra, 3000-602 Coimbra, Portugal; Department of Cardiothoracic Surgery and Thoracic Organ Transplantation, Unidade Local de Saúde de Coimbra, 3004-561 Coimbra, Portugal; Department of Pediatric Cardiology, Referral Center for Congenital Cardiac Defects, Unidade Local de Saúde de Coimbra, 3000-602 Coimbra, Portugal

## Introduction

Infective endocarditis (IE), though frequently linked to major structural heart defects, also poses a risk to individuals with minor congenital anomalies, such as bicuspid aortic valves. These patients face a substantially higher risk of IE compared to the general population,^1^ warranting clinical follow-up.

## Case presentation

**Figure 1:**
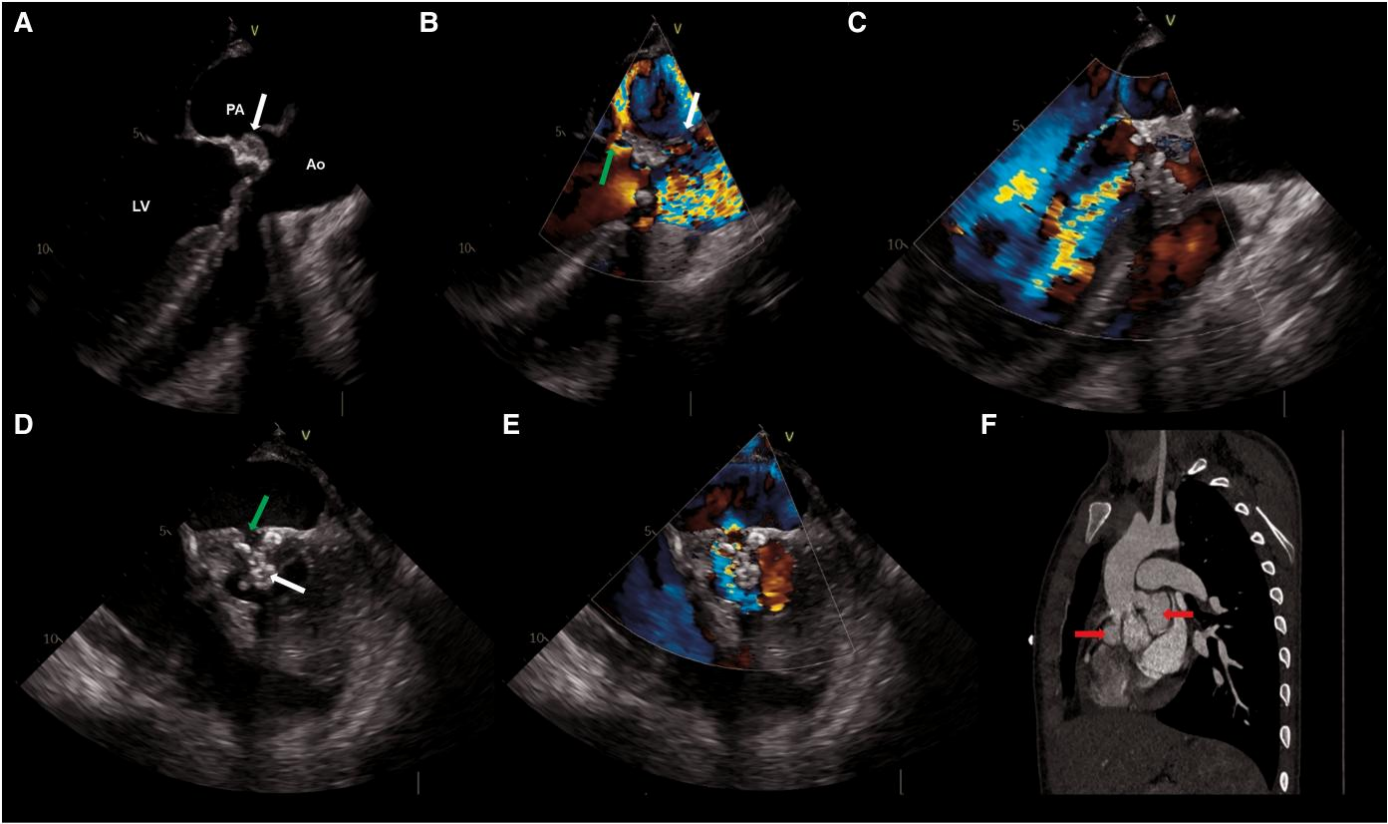
Transoesophageal echocardiogram and thoracic computed tomography scan. (*A*) Mid-oesophageal long-axis transoesophageal echocardiogram view showing large vegetations (white arrow) on the aortic valve leaflets and an associated sinus of Valsalva pseudoaneurysm. (*B*) Colour Doppler in the same transoesophageal echocardiogram view demonstrating turbulent aortic valve flow and the pseudoaneurysm (white arrow) draining into the left ventricle (green arrow). (*C*) Colour Doppler in the same transoesophageal echocardiogram view revealing severe aortic insufficiency during diastole. (*D*) Mid-oesophageal short-axis transoesophageal echocardiogram view highlighting vegetations (white arrow) on a bicuspid aortic valve and a large sinus of Valsalva pseudoaneurysm (green arrow). *(E)* Colour Doppler in the short-axis transoesophageal echocardiogram view showing aortic regurgitation and flow directed into the pseudoaneurysm. (*F*) Reconstructed sagittal computed tomography image of the aortic root demonstrating significant distortion and the presence of two pseudoaneurysms (red arrows), collectively forming the ‘garlic sign’. Label: Ao, ascending aorta; LV, left ventricle; PA, pseudoaneurysm.

We present the case of a 17-year-old male with a known history of a bicuspid aortic valve and mild regurgitation, who presented with a 3-month history of progressive fatigue, night sweats, myalgia, and significant weight loss, dropping from 83 to 70 kg. Symptoms began approximately 4 weeks after undergoing a dental procedure. The diagnosis of IE was assumed based on the presence of large vegetations on the aortic valve observed in a transthoracic echocardiogram and positive blood cultures for *Streptococcus gordonii*. A transoesophageal echocardiogram further confirmed the presence of severe aortic regurgitation (*Figure 1C*) and stenosis, as well as rupture of the sinuses of Valsalva, forming several pseudoaneurysms,^2^ one of which draining into the left ventricle (*Figure 1A*, *B*, *D*, and *E*). An abdominal ultrasound also identified splenic septic *emboli*. Empiric antimicrobial therapy with ceftriaxone, gentamicin, and ampicillin was started and later changed to ceftriaxone monotherapy. Despite some clinical and analytical improvement, the patient’s severe valve damage persisted. A thoracic computed tomography confirmed the presence of three pseudoaneurysms at the aortic root valve (*Figure 1F*), while brain magnetic resonance imaging revealed a cerebral abscess with associated microhaemorrhage. On Day 18 of treatment, the patient underwent a Bentall–DeBono procedure,^3^ with prosthetic valve replacement, complicated by complete atrioventricular block, requiring pacemaker implantation. Six weeks post-operatively, the patient was discharged on warfarin. During the most recent evaluation, the patient was asymptomatic and had regained his usual weight, reaching 81 kg. The data underlying this article cannot be publicly shared to protect the privacy of the patient described. Additional details may be provided upon reasonable request to the corresponding author.

## Conclusion

This case highlights the importance of vigilance for IE in patients with minor congenital heart defects and underscores the crucial role of multimodality imaging in both the diagnosis and management of complex cases. Advanced imaging techniques were instrumental in guiding clinical decision-making, reinforcing their value in optimizing patient outcomes.

**Consent:** Written informed consent was obtained from the patient’s legal representative, his mother.

**Funding:** This research received no specific grant from any funding agency, commercial or not-for-profit sectors.

## Data Availability

The data underlying this article will be shared on reasonable request to the corresponding author.
